# Storage Temperature Alters the Expression of Differentiation-Related Genes in Cultured Oral Keratinocytes

**DOI:** 10.1371/journal.pone.0152526

**Published:** 2016-03-29

**Authors:** Tor Paaske Utheim, Rakibul Islam, Ida G. Fostad, Jon R. Eidet, Amer Sehic, Ole K. Olstad, Darlene A. Dartt, Edward B. Messelt, May Griffith, Lara Pasovic

**Affiliations:** 1 Department of Medical Biochemistry, Oslo University Hospital, Oslo, Norway; 2 Department of Oral Biology, Faculty of Dentistry, University of Oslo, Oslo, Norway; 3 Department of Ophthalmology, Vestre Viken HF Trust, Drammen, Norway; 4 Faculty of Health Sciences, National Centre for Optics, Vision and Eye Care, Buskerud and Vestfold University College, Kongsberg, Norway; 5 Department of Ophthalmology, Oslo University Hospital, Oslo, Norway; 6 Schepens Eye Research Institute, Massachusetts Eye and Ear, Harvard Medical School, Boston, Massachusetts, United States of America; 7 Department of Clinical and Experimental Medicine, Linköping University, Linköping, Sweden; 8 Faculty of Medicine, University of Oslo, Oslo, Norway; University of Alabama at Birmingham, UNITED STATES

## Abstract

**Purpose:**

Storage of cultured human oral keratinocytes (HOK) allows for transportation of cultured transplants to eye clinics worldwide. In a previous study, one-week storage of cultured HOK was found to be superior with regard to viability and morphology at 12°C compared to 4°C and 37°C. To understand more of how storage temperature affects cell phenotype, gene expression of HOK before and after storage at 4°C, 12°C, and 37°C was assessed.

**Materials and Methods:**

Cultured HOK were stored in HEPES- and sodium bicarbonate-buffered Minimum Essential Medium at 4°C, 12°C, and 37°C for one week. Total RNA was isolated and the gene expression profile was determined using DNA microarrays and analyzed with Partek Genomics Suite software and Ingenuity Pathway Analysis. Differentially expressed genes (fold change > 1.5 and *P* < 0.05) were identified by one-way ANOVA. Key genes were validated using qPCR.

**Results:**

Gene expression of cultures stored at 4°C and 12°C clustered close to the unstored control cultures. Cultures stored at 37°C displayed substantial change in gene expression compared to the other groups. In comparison with 12°C, 2,981 genes were differentially expressed at 37°C. In contrast, only 67 genes were differentially expressed between the unstored control and the cells stored at 12°C. The 12°C and 37°C culture groups differed most significantly with regard to the expression of differentiation markers. The Hedgehog signaling pathway was significantly downregulated at 37°C compared to 12°C.

**Conclusion:**

HOK cultures stored at 37°C showed considerably larger changes in gene expression compared to unstored cells than cultured HOK stored at 4°C and 12°C. The changes observed at 37°C consisted of differentiation of the cells towards a squamous epithelium-specific phenotype. Storing cultured ocular surface transplants at 37°C is therefore not recommended. This is particularly interesting as 37°C is the standard incubation temperature used for cell culture.

## Introduction

The stem cells of the cornea are located in the periphery, in a region known as the limbus. Limbal stem cells can be destroyed by a multitude of diseases, including certain autoimmune diseases and genetic conditions [1]. These cells can also be damaged by external factors, such as chemical or thermal burns, ultraviolet radiation, and infections (e.g. trachoma). Contingent upon the extent of damage to limbal stem cells, various clinical presentations of limbal stem cell deficiency (LSCD) may develop. In the most serious cases, patients may become blind and experience substantial pain.

In 1997, LSCD was for the first time successfully treated by transplantation of *ex vivo* cultured limbal stem cells [2]. In unilateral LSCD, autologous limbal stem cells can be harvested from the contralateral healthy cornea, but this is generally not feasible in bilateral LSCD, which is by far the most common form. If allogeneic limbal stem cells are applied, immunosuppression, which can have severe adverse effects [3], is required at least for a certain period of time [1]. This has urged researchers to the search for alternative autologous cell sources.

**Fig 1:**
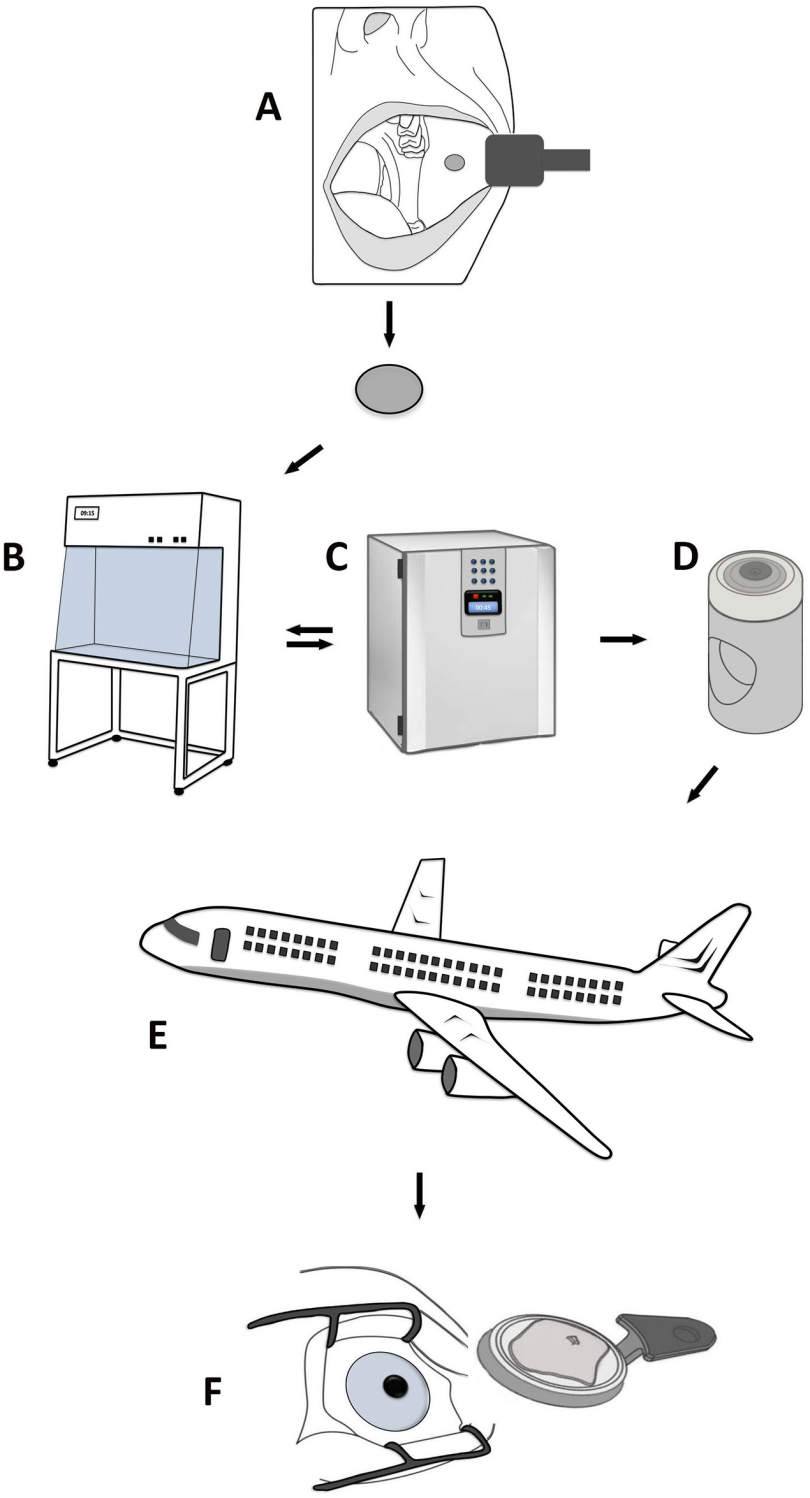
Possible steps in the treatment of limbal stem cell deficiency. An oral mucosa biopsy is removed from the mouth (A) and sent to a laboratory (B, C, D). Oral keratinocytes are then cultured in an incubator for six days before the generated cell sheet is transferred to a sealed storage container where it can be preserved for up to one week. This allows the cultured tissue to be returned to the patient (E) for transplantation onto the diseased eye (F). Courtesy of Amer Sehic, Department of Oral Biology, University of Oslo.

In 2004, oral keratinocytes were shown to be effective for treating LSCD in humans [4, 5]. Since then, there have been 20 clinical reports confirming their potential to treat LSCD [6]. Except for conjunctival cells, oral keratinocytes are the only non-limbal cell type that has been used clinically. Accumulating evidence of the rationale for transplanting cultured oral keratinocytes in LSCD substantiates the need to make this regenerative medicine technology available worldwide. Currently, the treatment is restricted to a few centers of expertise [6]. Increasingly stricter regulations for cell therapy will likely lead to the centralization of culture units [7]. Centralization requires effective transportation strategies [8], which calls for a practical method for storage of cultured cells outside the incubator (Fig 1).

Storage in a small sealed container for some days offers a number of advantages. These include: 1) sufficient time for phenotypic assessment of the cultured transplants prior to surgery, which has become increasingly important with recent knowledge about the critical role of the phenotype of transplants for good clinical outcome [9]; 2) microbiological assessment after aspiration of a storage medium sample from the septum of the hermetically sealed storage container; 3) increased flexibility for the surgeon in scheduling surgeries, which may be convenient if unforeseen factors related to the patient or cultured cells should occur [10, 11], and importantly; 4) transportation of transplants to reach eye clinics worldwide.

In a previous study, one-week storage of cultured human oral keratinocytes (HOK) at 12°C was superior with regard to viability and morphology compared to storage at 4°C and 37°C [12]. In the present study, we have used genome-wide analysis of gene expression to: 1) investigate whether differences in temperature following one-week storage result in phenotypic changes, and 2) explore potential mechanisms behind these differences. In the previous study, phenotype was analysed by immunocytochemistry and found to be preserved at both 4°C and 12°C, but not at 37°C [12]. Based on this finding, we hypothesize that cells stored at 37°C show substantial differences in gene expression compared to cells stored at 12°C.

## Materials and Methods

First passage normal HOK, oral keratinocyte medium (OKM), oral keratinocyte growth supplement (OKGS), and penicillin/streptomycin solution (P/S) were purchased from ScienCell Research Laboratories (San Diego, Carlsbad, CA, USA). Nunclon Δ-Cell culture flasks and pipettes were purchased from VWR International (West Chester, PA, USA). The Minimum Essential Medium (MEM) was obtained from Invitrogen (Carlsbad, CA, USA). Phosphate-buffered saline (PBS), 4-(2-hydroxyethyl)-1-piperazineethanesulfonic acid (HEPES), and sodium bicarbonate were all purchased from Sigma-Aldrich (St. Louis, MO). RNeasy Plus Mini Kit and the QIAzol Lysis Reagent were provided by Qiagen (Hilden, Germany).

### Culture and Storage of Human Oral Keratinocytes

Human oral keratinocytes were grown to confluence in T25 cell culture flasks in complete OKM (made by adding 5 mL OKGS and 5 mL P/S to 500 mL OKM), in a 37°C humidified incubator with 5% CO_2_ supply. The HOK were cultured in the dark, and the culture medium was changed every other day. All cells were cultured for six days. Control cells were immediately processed for analysis, while the rest were randomized to storage at either 4°C, 12°C or 37°C. These were stored for one week before being analyzed.

On day six of culture, when confluent cultures were obtained, the OKM was removed and the cultures were rinsed with PBS before adding the storage medium. The storage medium consisted of 70 mL MEM, 25 mM HEPES, 600 mg/L sodium bicarbonate, and 50 *μ*g/mL gentamycin (hereafter named MEM). The screw caps of the T-25 flasks were tightened to reduce air exchange and evaporation. The cultures were randomized for storage at three temperatures (4°C, 12°C, and 37°C) for one week. Cells cultured for six days, but not subjected to storage, served as controls in all experiments. Custom-built storage cabinets with a very small standard deviation (±0.4°C) for the set temperatures were used for regulating temperature during storage [13]. The temperature inside each storage container was monitored throughout all experiments. The storage cabinets were kept in a cold room maintaining an ambient temperature of 4°C. Each cabinet was equipped with a light bulb functioning as a heater, which increased the temperature inside the box from the ambient room temperature (4°C) to the desired storage temperature. The light bulbs were continuously regulated by a highly sensitive thermometer, and the storage containers were equipped with a small fan that ensured a homogeneous temperature inside the box by circulating the air. The light bulbs were separated from the cells by dark walls, which ensured that the cells were not directly exposed to light, and minimized indirect light exposure. However, we cannot exclude the possibility that cells stored at higher temperatures (12°C and 37°C) were to some extent exposed to the light.

### Isolation of RNA

Cultured HOK stored for one week at 4°C, 12°C, and 37°C, and control cultures that had not been subjected to storage, were rinsed with PBS and directly lysed with QIAzol Lysis Reagent. According to the manufacturer’s protocol, the fractions of total RNA were isolated using miRNeasy Mini Kit (Qiagen). The concentrations of purified RNA were assayed using a Nanodrop ND-1000 Spectrophotometer (Thermo Fisher Scientific, Wilmington, DE, USA). This yielded RNA fractions exhibiting absorbance ratios—A_260/280_ and A_260/230_ –of at least 1.8 and 2.0, respectively. The quality of RNA in solutions was assessed using the Agilent-Bioanalyzer 2100 System and RNA 6000 Nano Assay (Agilent Technologies, Santa Clara, CA, USA). All solutions used had RNA integrity number (RIN) values of > 8.5.

### Microarray Analysis

The Affymetrix GeneChip Human Gene 1.0 ST Microarrays (Affymetrix, Santa Clara, CA, USA) used in this study included approximately 28,000 gene transcripts. Microarray analysis was carried out in triplicate using cultured HOK stored for one week at 4°C, 12°C, and 37°C. Unstored control cultures served as control. Preparation of complementary DNA (cDNA) was carried out using GeneChip HT One-Cycle cDNA Synthesis Kit (Affymetrix). Each of three microarrays was hybridized with cDNA prepared from 150 ng of total RNA from each resulting solution. Biotinylated and fragmented single stranded cDNAs were hybridized to the GeneChips. The arrays were washed and stained using FS-450 fluidics station (Affymetrix).

Signal intensities were detected by Hewlett Packard Gene Array Scanner 3000 7G (Hewlett Packard, Palo Alto, CA, USA). The scanned images were processed using the AGCC (Affymetrix GeneChip Command Console) software and the CEL files were imported into Partek Genomics Suite software (Partek, Inc. MO, USA). The Robust Multichip Analysis (RMA) algorithm was applied to generate signal values and normalization. Gene transcripts with maximal signal values of less than 32 across all arrays were removed to filter for low and non-expressed genes, reducing the number of gene transcripts to 17,684. For expression comparisons of different groups, profiles were compared using a one-way ANOVA model. The results were expressed as fold changes (FC) with corresponding *P-*values.

### Bioinformatic Analysis

Bioinformatic analysis using Ingenuity Pathways Analysis (IPA) (Ingenuity Inc, IL) was carried out to find molecular and cellular functions and canonical pathways that were significantly associated with differentially expressed genes. Briefly, the data set containing gene identifiers and corresponding fold changes and *P-*values was uploaded onto the web-delivered application and each gene identifier was mapped to its corresponding gene object in the Ingenuity Pathways Knowledge Base (IPKB). Functional analysis identified the biological functions and/or diseases that were significantly associated with the data sets. Fisher’s exact test was performed to calculate a *P*-value determining the probability that each biological function and/or disease assigned to the data set was due to chance alone. The data sets were mined for significant pathways with the IPA library of canonical pathways, using IPA generated networks as graphical representations of the molecular relationships between genes and gene products.

### Validation of Microarray Results by Quantitative Real-Time PCR

The differential gene expression data were validated for selected transcripts using TaqMan^®^ Gene Expression Assays and the Applied Biosystems^®^ViiA^™^ 7 Real-Time PCR system (Applied Biosystems, Life Technologies, Carlsbad, CA, USA). The genes encoding heat shock 22kDa protein 8 (HSPB8), tumor protein p63 (TP63), and keratin 10 (KRT10) were selected for validation. Briefly, 200 ng of total RNA was reverse transcribed using qScript^™^ cDNA Super Mix (Quanta Biosciences Gaithersburg, MD) following the manufacturer’s instructions. After completion of cDNA synthesis, 1/10th of the first strand reaction was used for PCR amplification. A total amount of 9 μl of diluted cDNA (diluted in H_2_O), 1 μl of selected primer/ probes TaqMan^®^ Gene Expression Assays (Life Technologies), and 10 μl TaqMan^®^ Universal Master Mix (Life Technologies) were used, as per the manufacturer’s instructions. Transuducin-like enhancer of split 1 (TLE1) was used as an endogenous control due to the low coefficient of variation (CV) (0.444) in the Affymetrix study. Each gene was run in duplicates. TaqMan^®^ Gene Expression Assays (Life Technologies) used assays detecting HSPB8 (HSPB8-Hs00205056_m1), TP63 (TP63-Hs00978343_m1), KRT10 (KRT10-Hs01043114_g1), and TLE1 (Hs00270768_m1).

*P*-values were calculated using Student's t-test in Microsoft Excel (Redmond, WA, USA) using delta Ct-values. Normalized target gene expression levels were calculated using the formula: 2^(–ΔΔCt).

## Results

### Global Perspective of Microarray Results

**Fig 2:**
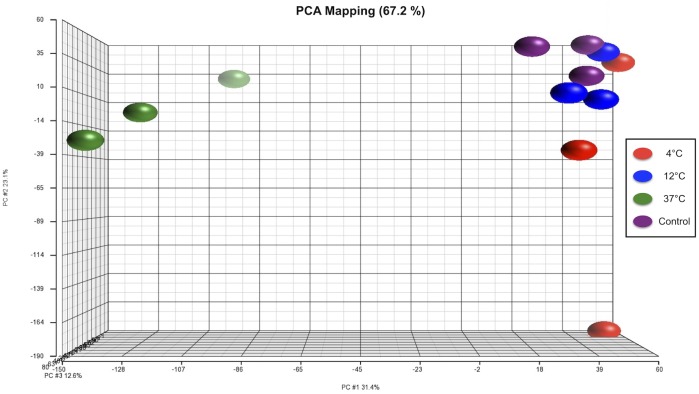
Principal component analyses demonstrated clustering of the gene expression of unstored cultures (violet) and cultures stored for one week at 4°C (red) and 12°C (blue). In contrast, gene expression of cultures stored at 37°C (green) showed a distant clustering compared to the other experimental groups.

**Fig 3:**
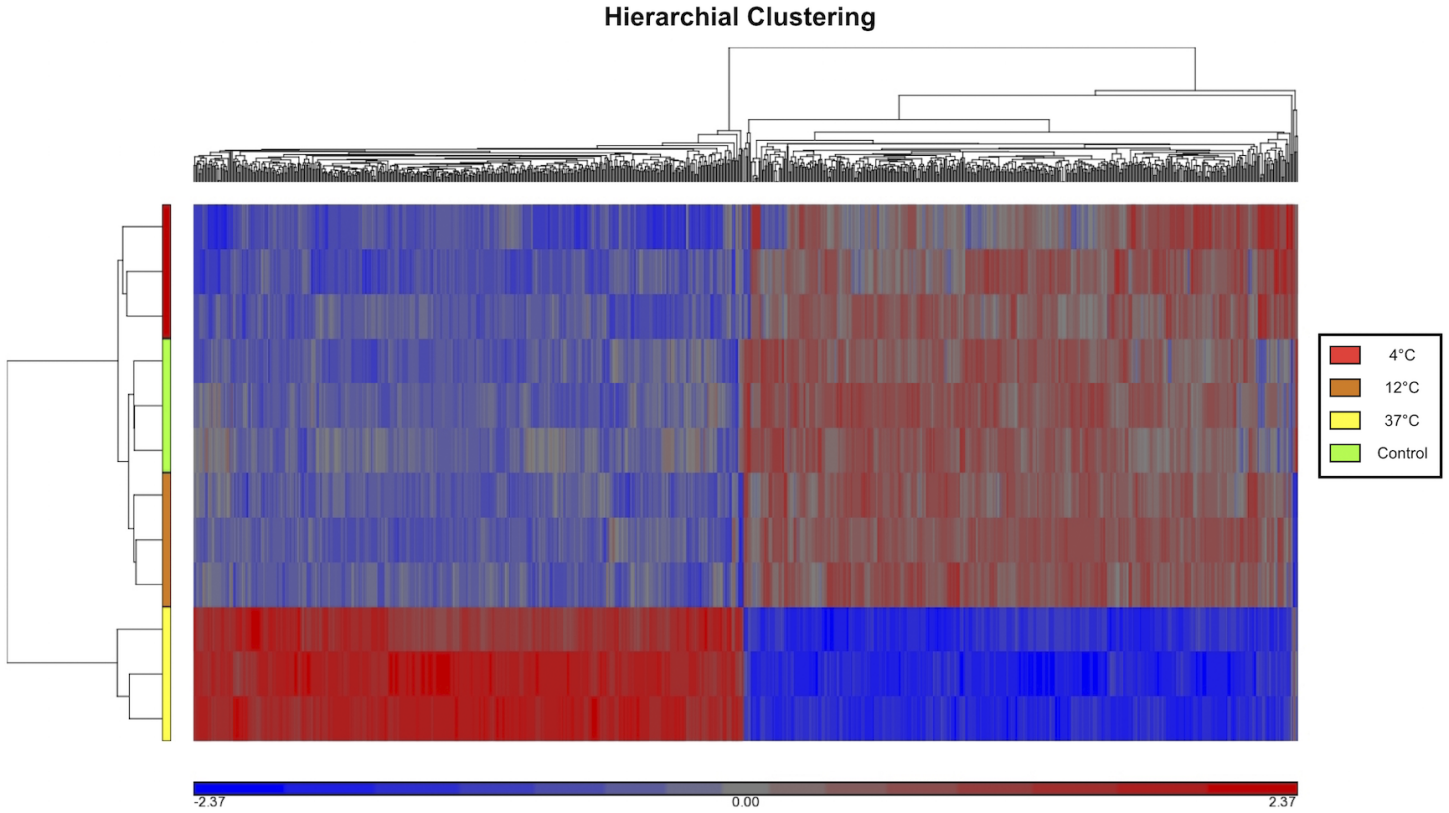
Hierarchical cluster analysis visualizing differences in gene expression between the cultures stored at 37°C and the remaining experimental groups (unstored cultures and cultures stored for one week at 4°C and 12°C).

Gene expression of cultures stored at 4°C and 12°C were clustered close to those of fresh cultures that had not been subjected to storage (control group) (Fig 2). Cultures stored at 37°C displayed substantial change in gene expression compared to the other groups (Fig 3). In comparison with 12°C, 2,981 genes were differentially expressed at 37°C (Table 1). In contrast, only 67 and 117 genes were differentially expressed when comparing the 12°C group to the control and the 4°C group, respectively. While only 67 genes were differentially regulated at 12°C compared to control cells, almost twice as many (124) were differentially regulated at 4°C compared to the control. Given the relatively small differences between the control, 4°C and 12°C but large differences between 12°C and 37°C, we have chosen to direct our focus primarily on the differential gene expression between 12°C and 37°C.

**Table 1:** Number of differentially expressed genes (*P* < 0.05; FC > 1.5).

Comparison	Number of genes changed >1.5-fold	Number of genes downregulated (% of total)	Number of genes upregulated (% of total)
Control vs. 12°C	67	25 (37.3%)	42 (62.7%)
4°C vs. 12°C	117	59 (50.4%)	58 (49.6%)
37°C vs. 12°C	2981	1486 (49.8%)	1495 (50.2%)
4°C vs. Control	124	76 (61.3%)	48 (38.7%)

### The Most Differentially Regulated Genes

Repetin (RPTN) was the most differentially regulated gene, with a 136.9-fold upregulation at 37°C compared to 12°C. Repetin is a constituent of the epidermal differentiation complex and functions in the cornified cell envelope formation [14]. Desmoglein (DSG1) was the second most upregulated gene at 37°C compared to 12°C, with a 94.6-fold upregulation at this temperature. It is a constituent of the desmosome, providing cell-cell adhesion [15]. Keratinocyte differentiation-associated protein (KDAP) was upregulated 54.7-fold at 37°C compared to 12°C. It is a regulatory protein of keratinocyte differentiation and influences the stratification of epithelia [16, 17]. Keratin 10 (KRT10), involved in the differentiation of human oral keratinocytes [18], was upregulated 45.6-fold at 37°C compared to 12°C. Lipase K (LIPK), a gene which is highly specific for the last step of keratinocyte differentiation [19], was upregulated 43.9-fold at 37°C compared to 12°C. Cornulin (CRNN), another marker of late epidermal differentiation [20], was upregulated 43.2-fold at 37°C compared to 12°C (Table 2). Hence, the most differentially regulated proteins at 37°C compared to 12°C are directly associated with differentiation of epithelia.

**Table 2:** Top ten upregulated genes during storage.

Gene Symbol	Gene Description	Affymetrix ID	*P*-value	Fold Change
**Control vs. 12°C**				
mir-31	microRNA 31	8160439	2.31E-03	7.57
LCE3D	late cornified envelope 3D	7920185	8.65E-03	6.91
mir-503	microRNA 503	8175261	1.64E-03	6.84
MIR205HG	MIR205 host gene (non-protein coding)	7909422	2.71E-02	4.22
IFNE	interferon, epsilon	8160435	2.13E-03	3.85
mir-21	microRNA 21	8008885	1.61E-02	3.53
TAS2R4	taste receptor, type 2, member 4	8136645	2.67E-02	3.27
VPS29	vacuolar protein sorting 29 homolog (S. cerevisiae)	7966343	3.73E-03	3.17
TRIM52	tripartite motif containing 52	8110666	1.61E-03	3.14
mir-24	microRNA 24–1	8034694	4.50E-02	2.92
**4°C vs. 12°C**				
mir-31	microRNA 31	8160439	2.00E-03	7.95
HIST1H4B	histone cluster 1, H4b	8124385	8.03E-04	5.20
SNORA74A	small nucleolar RNA, H/ACA box 74A	8108420	1.68E-03	5.03
RPSA	ribosomal protein SA	8078918	2.05E-02	4.99
mir-21	microRNA 21	8008885	4.87E-03	4.95
TAS2R4	taste receptor, type 2, member 4	8136645	6.94E-03	4.83
HIST2H4B	histone cluster 2, H4b	8124521	7.31E-03	4.39
C9orf3	chromosome 9 open reading frame 3	8156571	3.64E-02	3.79
HIST1H4C	histone cluster 1, H4c	8117368	2.81E-02	3.69
HIST1H4A	histone cluster 1, H4a	8117334	1.47E-02	3.60
**37°C vs. 12°C**				
RPTN	repetin	7920146	1.84E-07	136.92
DSG1	desmoglein 1	8020724	7.86E-07	94.61
KDAP	keratinocyte differentiation-associated protein	8036072	2.13E-05	54.65
KRT10	keratin 10	8015104	9.32E-05	45.63
LIPK	lipase, family member K	7928994	2.46E-05	43.89
CRNN	cornulin	7920178	8.85E-04	43.19
TMPRSS11B	transmembrane protease, serine 11B	8100701	3.54E-03	38.52
SPINK7	serine peptidase inhibitor, Kazal type 7 (putative)	8109049	5.78E-03	36.93
MUC15	mucin 15, cell surface associated	7947156	8.52E-08	36.71
MAP2	microtubule-associated protein 2	8047926	1.47E-07	34.06
**4°C vs. control**				
MT-TE	mitochondrially encoded tRNA glutamic acid	8165707	3.67E-02	8.17
SNORA52	small nucleolar RNA. H/ACA box 52	7937483	3.84E-02	5.37
SNORA74A	small nucleolar RNA. H/ACA box 74A	8108420	4.29E-03	5.13
SNORD14E	small nucleolar RNA. C/D box 14E	7952335	8.97E-03	4.98
RNU4-2	RNA. U4 small nuclear 2	7967028	4.32E-02	4.92
RNA5SP242	RNA. 5S ribosomal pseudogene 242	8135943	3.77E-02	4.52
SCARNA9L	small Cajal body-specific RNA 9-like	8171758	2.34E-03	3.45
HIST1H4J	histone cluster 1. H4j	8117598	4.17E-02	3.45
RNA5SP191	RNA. 5S ribosomal pseudogene 191	8107857	4.44E-02	3.40
EIF4A2	eukaryotic translation initiation factor 4A2	8084708	3.40E-02	3.36

Melatonin receptor 1A (MTNR1A) was significantly upregulated in the 12°C storage group compared to all other groups: 5.4-fold compared to control cultures, 4.8-fold compared to 4°C and 5.9-fold compared to 37°C (Table 3). It was the single most upregulated gene when comparing the 12°C group to the control and the 4°C group. Expression of MTNR1A in the skin is modified by several factors, including UVB exposure [21]. A significant association between MTNR1A polymorphisms and oral carcinogenesis has been demonstrated [22], and MTNR1A has been designated a putative tumor suppressor [23].

**Table 3:** Top ten downregulated genes during storage.

Gene Symbol	Gene Description	Affymetrix ID	*P*-value	Fold Change
**Control vs. 12°C**				
MTNR1A	melatonin receptor 1A	8104074	4.71E-04	-5.41
GADD45B	growth arrest and DNA-damage-inducible, beta	8024485	3.80E-04	-3.61
RNU11	RNA, U11 small nuclear	7899502	1.60E-02	-2.55
RPL13A	ribosomal protein L13a	8030364	8.82E-03	-2.45
CSRNP1	cysteine-serine-rich nuclear protein 1	8086330	1.67E-02	-2.41
C9orf131	chromosome 9 open reading frame 131	8160912	4.45E-02	-2.38
NXF1	nuclear RNA export factor 1	7948839	1.11E-03	-2.30
VTRNA1-3	vault RNA 1–3	8108631	1.14E-02	-2.30
MAB21L3	mab-21-like 3 (C. elegans)	7904244	8.77E-03	-2.21
IFRD1	interferon-related developmental regulator 1	8135514	1.71E-02	-2.19
**4°C vs. 12°C**				
MTNR1A	melatonin receptor 1A	8104074	7.56E-04	-4.80
GADD45B	growth arrest and DNA-damage-inducible, beta	8024485	1.52E-03	-2.81
mir-181	microRNA 181a-1	7923173	1.95E-02	-2.57
CSRNP1	cysteine-serine-rich nuclear protein 1	8086330	2.36E-02	-2.25
FBXO32	F-box protein 32	8152703	2.50E-02	-2.25
NXF1	nuclear RNA export factor 1	7948839	1.87E-03	-2.15
TGM2	transglutaminase 2	8066214	3.06E-01	-2.07
MAB21L3	mab-21-like 3 (C. elegans)	7904244	1.59E-02	-2.02
CCDC80	coiled-coil domain containing 80	8089544	4.72E-02	-1.96
IFI35	interferon-induced protein 35	8007446	4.02E-03	-1.85
**37°C vs. 12°C**				
TFPI2	tissue factor pathway inhibitor 2	8141016	3.60E-08	-13.24
FKBP5	FK506 binding protein 5	8125919	1.62E-06	-12.53
ANPEP	alanyl (membrane) aminopeptidase	7991335	5.25E-07	-12.44
CDC20	cell division cycle 20	7900699	7.36E-03	-12.19
RNU5D-1	RNA, U5D small nuclear 1	7915592	3.90E-04	-11.70
DTL	denticleless E3 ubiquitin protein ligase homolog (Drosophila)	7909568	2.21E-02	-11.52
ANGPTL4	angiopoietin-like 4	8025402	2.70E-03	-11.15
PLK1	polo-like kinase 1	7994109	1.56E-02	-10.23
TPX2	TPX2, microtubule-associated	8061579	2.39E-03	-10.23
PLAT	plasminogen activator, tissue	8150509	1.53E-03	-9.77
**4°C vs. control**				
LCE3D	late cornified envelope 3D	7920185	8.89E-03	-5.94
LCE3E	late cornified envelope 3E	7920182	2.47E-02	-3.37
MIR222	microRNA 222	8172268	1.52E-02	-3.03
DEFB103A	defensin beta 103A	8149172	3.12E-02	-3.00
MIR503	microRNA 503	8175261	4.12E-02	-2.72
KPRP	keratinocyte proline rich protein	7905515	5.41E-03	-2.67
RNA5SP82	RNA. 5S ribosomal pseudogene 82	7925701	4.75E-02	-2.44
MIR181B1	microRNA 181b-1	7923173	2.38E-02	-2.40
VPS29	VPS29 retromer complex component	7966343	2.06E-02	-2.34
SLITRK6	SLIT and NTRK like family member 6	7972239	8.89E-03	-2.23

Cysteine-serine-rich nuclear protein 1 (CSRNP1) was upregulated 2.4-fold after storage at 12°C compared to control cultures (Table 3). This result is in line with recent findings from our research group, indicating that CSRNP1 is the second most upregulated gene (12.7-fold increase) in retinal pigment epithelial cells, when stored at 16°C compared to unstored cells (unpublished data).

Late cornified envelope 3D (LCE3D) was downregulated 5.9-fold at 4°C and 6.9-fold at 12°C compared to the control. Similarly, late cornified envelope 3E (LCE3E) and keratinocyte proline rich protein (KPRP) were downregulated 3.4-fold and 2.7-fold at 4°C compared to the control, respectively. Genes that code for late cornified envelope proteins are enriched and clustered within the epidermal differentiation complex [24, 25]. The relatively low differences in gene expression at 4°C and 12°C compared to the control stand in sharp contrast to the much greater changes observed when comparing 12°C cultures to 37°C.

### Expression of Differentiation Markers

**Fig 4:**
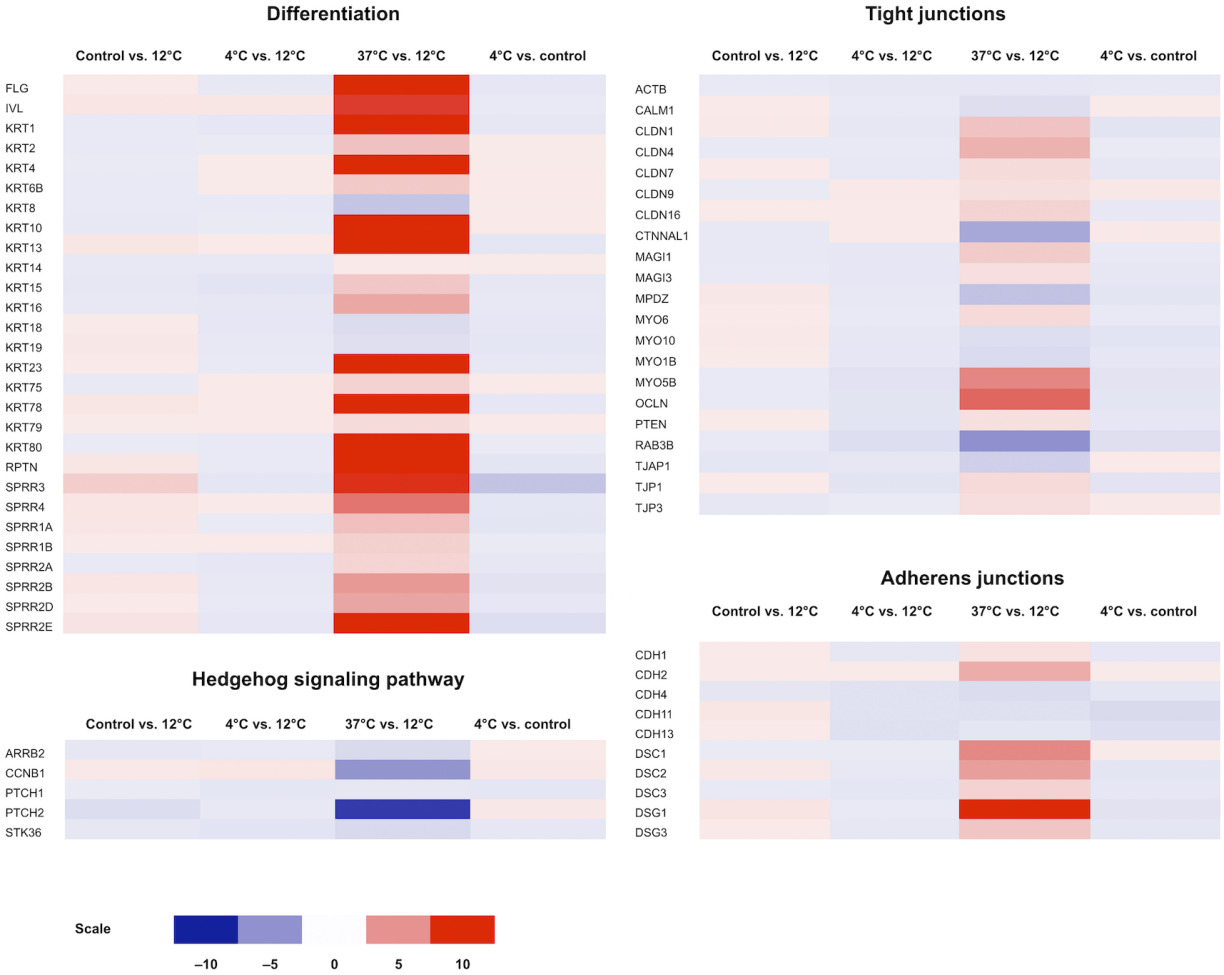
Heat map diagrams of a selection of the most important genes expressed by cultured human oral keratinocytes (HOK) related to differentiation, tight and adherens junctions, and the Hedgehog signaling pathway, respectively. The color scale illustrates the relative expression level of mRNAs: red color represents a high expression level; blue color represents a low expression level.

In addition to RPTN, KDAP, KRT10, LIPK, and CRNN, as presented in the previous section, the following genes associated with differentiation were upregulated: First, the oral mucosal differentiation marker keratin 4 (KRT4) [26] was upregulated 10.2-fold at 37°C storage compared to 12°C (Table 4 and Fig 4), indicative of a lower degree of differentiation in 12°C cultures. Second, keratin 6B, a specific marker of oral mucosal cells [26], was upregulated 2.5-fold at 37°C compared to 12°C cultures. Third, expression of keratin 19 (KRT19), a marker of undifferentiated cells [27], was 1.5-fold higher in cells stored at 12°C compared to those stored at 37°C (Table 4). Taken together, a total of 17 keratins were differentially regulated at 37°C compared to 12°C; 14 of these were upregulated.

**Table 4:** Differential regulation of genes in HOK cultures stored at 37°C compared to HOK cultures stored at 12°C.

Gene Symbol	Gene Description	Affymetrix ID	*P*-value	Fold Change
**Differentiation**				
FLG	filaggrin	7920165	1.71E-03	18.90
IVL	involucrin	7905533	5.74E-04	8.76
KRT1	keratin 1	7963491	3.51E-03	13.49
KRT2	keratin 2	7963479	1.85E-02	2.88
KRT4	keratin 4	7963534	4.66E-03	10.21
KRT6B	keratin 6B	7963406	1.76E-04	2.53
KRT8	keratin 8	7963567	4.64E-05	-2.74
KRT10	keratin 10	8015104	9.32E-05	45.63
KRT13	keratin 13	8015323	1.57E-04	26.79
KRT14	keratin 14	8015366	1.90E-01	1.10
KRT15	keratin 15	8015337	3.63E-03	2.64
KRT16	keratin 16	8015376	2.49E-04	4.11
KRT18	keratin 18	7969574	3.07E-02	-1.67
KRT19	keratin 19	8015349	3.96E-02	-1.50
KRT23	keratin 23 (histone deacetylase inducible)	8015133	6.84E-03	16.39
KRT75	keratin 75	7963396	1.32E-03	2.13
KRT78	keratin 78	7963555	5.24E-04	17.62
KRT79	keratin 79	7963545	3.75E-02	1.76
KRT80	keratin 80	7963333	2.32E-04	10.63
LIPM	lipase, familiy member M	7929003	2,28E-02	3.72
RPTN	repetin	7920146	1.84E-07	136.92
SPRR3	small proline-rich protein 3	7905548	2.11E-03	9.39
SPRR4	small proline-rich protein 4	7905536	9.45E-03	6.33
SPRR1A	small proline-rich protein 1A	7905544	6.29E-04	3.01
SPRR1B	small proline-rich protein 1B	7905553	1.12E-03	2.26
SPRR2A	small proline-rich protein 2A	7920205	1.77E-03	2.05
SPRR2B	small proline-rich protein 2B	7920210	5.78E-04	4.77
SPRR2D	small proline-rich protein 2D	7920196	1.42E-03	4.22
SPRR2E	small proline-rich protein 2E	7920214	9.27E-05	11.18
TP63	tumor protein p63	8084766	1,49E-03	-1.667
**Tight junctions**				
ACTB	actin, beta	8137979	7.57E-02	-1.16
CALM1 (includes others)	calmodulin 1 (phosphorylase kinase, delta)	8029831	1.73E-03	-1.54
CLDN1	claudin 1	8092726	8.59E-03	2.86
CLDN4	claudin 4	8133360	7.88E-05	3.65
CLDN7	claudin 7	8012126	2.05E-02	1.71
CLDN9	claudin 9	7992782	3.54E-04	1.53
CLDN16	claudin 16	8084788	4.67E-03	2.11
CTNNAL1	catenin (cadherin-associated protein), alpha-like 1	8163063	1.14E-03	-4.02
MAGI1	membrane associated guanylate kinase, WW and PDZ domain containing 1	8088602	3.27E-03	2.46
MAGI3	membrane associated guanylate kinase, WW and PDZ domain containing 3	7904106	5.09E-03	1.57
MPDZ	multiple PDZ domain protein	8160088	6.35E-05	-2.84
MYO6	myosin VI	8120783	2.30E-04	1.78
MYO10	myosin X	8111153	1.05E-02	-1.60
MYO1B	myosin IB	8047127	7.66E-05	-1.71
MYO5B	myosin VB	8023267	6.73E-05	5.57
OCLN	occludin	8105908	8.34E-05	6.95
PTEN	phosphatase and tensin homolog	7928959	6.68E-03	1.51
RAB3B	RAB3B, member RAS oncogene family	7916112	4.75E-07	-5.10
TJAP1	tight junction associated protein 1 (peripheral)	8119829	1.14E-03	-2.20
TJP1	tight junction protein 1	7986977	2.63E-03	1.81
TJP3	tight junction protein 3	8024687	2.52E-02	1.58
**Adherens junctions**				
CDH1	cadherin 1, type 1, E-cadherin (epithelial)	7996837	1.10E-02	1.42
CDH2	cadherin 2, type 1, N-cadherin (neuronal)	8022674	5.42E-03	3.90
CDH4	cadherin 4, type 1, R-cadherin (retinal)	8063796	8.11E-03	-1.66
CDH11	cadherin 11, type 2, OB-cadherin (osteoblast)	8001800	2.33E-01	-1.49
CDH13	cadherin 13	7997504	2.93E-01	-1.21
DSC1	desmocollin 1	8022728	3.30E-06	5.51
DSC2	desmocollin 2	8022711	1.86E-05	4.48
DSC3	desmocollin 3	8022692	3.36E-04	2.15
DSG1	desmoglein 1	8020724	7.86E-07	94.61
DSG3	desmoglein 3	8020762	8.10E-05	2.78
**Stress response**				
NOS1	nitric oxide synthase 1 (neuronal)	7966779	9.01E-05	-3.82
HMOX1	heme oxygenase (decycling) 1	8072678	2.73E-04	5.33
HSP90B1	heat shock protein 90kDa beta (Grp94), member 1	7958130	0.00589	-1.78
HSPA9	heat shock 70kDa protein 9 (mortalin)	8114455	0.0823	-1.26
HSPA1A/HSPA1B	heat shock 70kDa protein 1A	8118314	0.0262	2.31
HSPA4L	heat shock 70kDa protein 4-like	8097335	0.015	1.58
HSPB1	heat shock 27kDa protein 1	8133721	0.0207	2.00
HSPB8	heat shock 22kDa protein 8	7959102	3.64E-06	27.55
HSPD1	heat shock 60kDa protein 1 (chaperonin)	8058052	8.32E-04	-1.91
**Hedgehog signaling pathway**				
ARRB2	arrestin, beta 2	8003903	1.24E-02	-1.74
CCNB1	cyclin B1	8105828	7.61E-03	-4.93
PTCH1	patched 1	8162533	4.96E-01	-1.12
PTCH2	patched 2	7915612	7.45E-05	-8.64
STK36	serine/threonine kinase 36	8048381	2.97E-03	-1.78
**Cell apoptosis and death**				
ABL1	ABL proto-oncogene 1, non-receptor tyrosine kinase	8158725	6.51E-04	-1.74
AKT3	v-akt murine thymoma viral oncogene homolog 3	7925531	2.57E-02	-1.70
AKTIP	AKT interacting protein	8001410	2.35E-05	2.59
ATM	ATM serine/threonine kinase	7943620	3.39E-03	-1.74
BAG2	BCL2-associated athanogene 2	8120402	9.80E-04	-2.45
BCL6	B-cell CLL/lymphoma 6	8092691	5.68E-04	2.17
BCL9	B-cell CLL/lymphoma 9	7904907	4.92E-03	-1.57
CASP4	caspase 4, apoptosis-related cysteine peptidase	7951372	0.0104	1.66
CFLAR	CASP8 and FADD-like apoptosis regulator	8047381	2.59E-02	1.88
DAPK1	death-associated protein kinase 1	8156199	8.43E-04	4.41
GADD45B	growth arrest and DNA-damage-inducible, beta	8024485	5.94E-03	-2.26
IKBKE	inhibitor of kappa light polypeptide gene enhancer in B-cells, kinase epsilon	7909188	3.05E-03	1.72
IL1A	interleukin 1, alpha	8054712	1.33E-02	-1.67
IL1B	interleukin 1, beta	8054722	1.67E-02	-2.50
LIPH	lipase, member H	8092541	3.72E-05	13.68
MAP3K1	mitogen-activated protein kinase kinase kinase 1, E3 ubiquitin protein ligase	8105436	3.36E-05	2.03
MYC	v-myc avian myelocytomatosis viral oncogene homolog	8148317	1.33E-03	-1.66
MYD88	myeloid differentiation primary response 88	8078729	9.49E-04	1.89
PARP1	poly (ADP-ribose) polymerase 1	7924733	8.67E-03	-2.13
PPP3CB	protein phosphatase 3, catalytic subunit, beta isozyme	7934393	1.03E-02	-1.67
RIPK3	receptor-interacting serine-threonine kinase 3	7978312	1.35E-02	1.60
TNFRSF10A	tumor necrosis factor receptor superfamily, member 10a	8149762	4.81E-04	-1.53
TNFRSF10B	tumor necrosis factor receptor superfamily, member 10b	8149733	7.64E-03	-1.69
TNFRSF10D	tumor necrosis factor receptor superfamily, member 10d, decoy with truncated death domain	8149749	1.59E-02	-1.67
TNFRSF9	tumor necrosis factor receptor superfamily, member 9	7912145	7.42E-05	2.79
TNFSF10	tumor necrosis factor (ligand) superfamily, member 10	8092169	4.86E-04	4.66
TOP2A	topoisomerase (DNA) II alpha 170kDa	8014974	3.96E-02	-6.14
TP53BP2	tumor protein p53 binding protein 2	7924526	1.61E-02	1.58
**Squamous metaplasia**				
FLG	filaggrin	7920165	1.71E-03	18.90
IVL	involucrin	7905533	5.74E-04	8.76
MAPK1	mitogen-activated protein kinase 1	8074791	3.16E-01	1.23
MAPK3	mitogen-activated protein kinase 3	8000811	3.86E-01	1.20
MAPK7	mitogen-activated protein kinase 7	8005576	3.34E-02	1.40
MAPK8	mitogen-activated protein kinase 8	7927389	1.65E-01	1.15
MAPK9	mitogen-activated protein kinase 9	8116402	2.52E-01	-1.19
MAPK12	mitogen-activated protein kinase 12	8076962	5.05E-05	-2.39
TGM2	transglutaminase 2	8066214	5.79E-01	-1.47
TGM3	transglutaminase 3	8060432	3.02E-02	5.20
TGM5	transglutaminase 5	7988050	1.91E-02	5.95

Other structural markers of keratinocyte differentiation had also changed during storage at 37°C. Filaggrin (FLG), which aggregates keratin intermediate filaments in mammalian epidermis [28], was upregulated 18.9-fold at 37°C compared to 12°C. Involucrin (IVL), a marker of differentiated keratinocytes [17, 18, 27], was upregulated 8.8-fold in cells stored at 37°C compared to those stored at 12°C, indicating increased differentiation of cells stored at 37°C. The lipase M (LIPM) gene, closely related to lipase K and exclusively expressed in the epidermis [19], was upregulated 3.7-fold at 37°C compared to 12°C (Table 4).

The cornified cell envelope is an insoluble protein layer that provides barrier function to stratified squamous epithelial cells [29]. Small proline-rich proteins (SPRRs) are constituents of this structure, and their expression is restricted to terminally differentiating squamous cells [18, 30]. Eight SPRR genes were upregulated between 2- and 11-fold at 37°C compared to 12°C, further indicating a more differentiated phenotype of cells stored at this temperature (Table 4). Apart from a 1.7-fold downregulation of TP63, a marker of undifferentiated cells, at 37°C compared to 12°C, few stem cell markers seemed to be affected by storage temperature. Neither OCT-4, FGF2, nor Nanog were differentially expressed at 12°C compared to 37°C, suggesting no significant impact of storage temperature on these stem cell related genes.

### Regulation of Cell-Cell Contact

Identified claudins (CLDN) 1, 4, 7, 9, and 16 were upregulated between 1.5 and 3.6-fold at 37°C compared to 12°C. Genes encoding tight junction proteins 1 (TJP1) and 3 (TJP3) were both upregulated 1.8 and 1.6-fold at 37°C compared to 12°C, respectively (Table 4 and Fig 4). These changes indicate an increased synthesis of tight junctions in cells stored at 37°C.

Regulatory changes of the constituents of the desmosomal adherens junction were noted (Table 4 and Fig 4). Desmosomes are intercellular junctions that link the intermediate filaments of the cytoskeleton of neighboring epithelial cells and consist of desmocollins, desmogleins, and cadherins [15]. Desmocollins (DSC) 1, 2, and 3 were upregulated 5.5, 4.5, and 2.2-fold at 37°C compared to 12°C, respectively. Loss of function of these genes is associated with skin barrier defects [31] and metastasis of cancer cells [32]. Desmocollin 3 has been described as a tumor suppressor of several types of cancer [33–36]. Desmogleins 1 and 3 were upregulated 94.6-fold and 2.8-fold, respectively. Of the many identified cadherins in our material, only cadherins 2 and 4 were differentially regulated, with a 3.9-fold upregulation and a 1.7-fold downregulation at 37°C, respectively (Table 4). Our findings point in the direction of increased adherence between cells stored at 37°C compared to other temperatures.

### Regulation of Cellular Stress Responses

**Fig 5:**
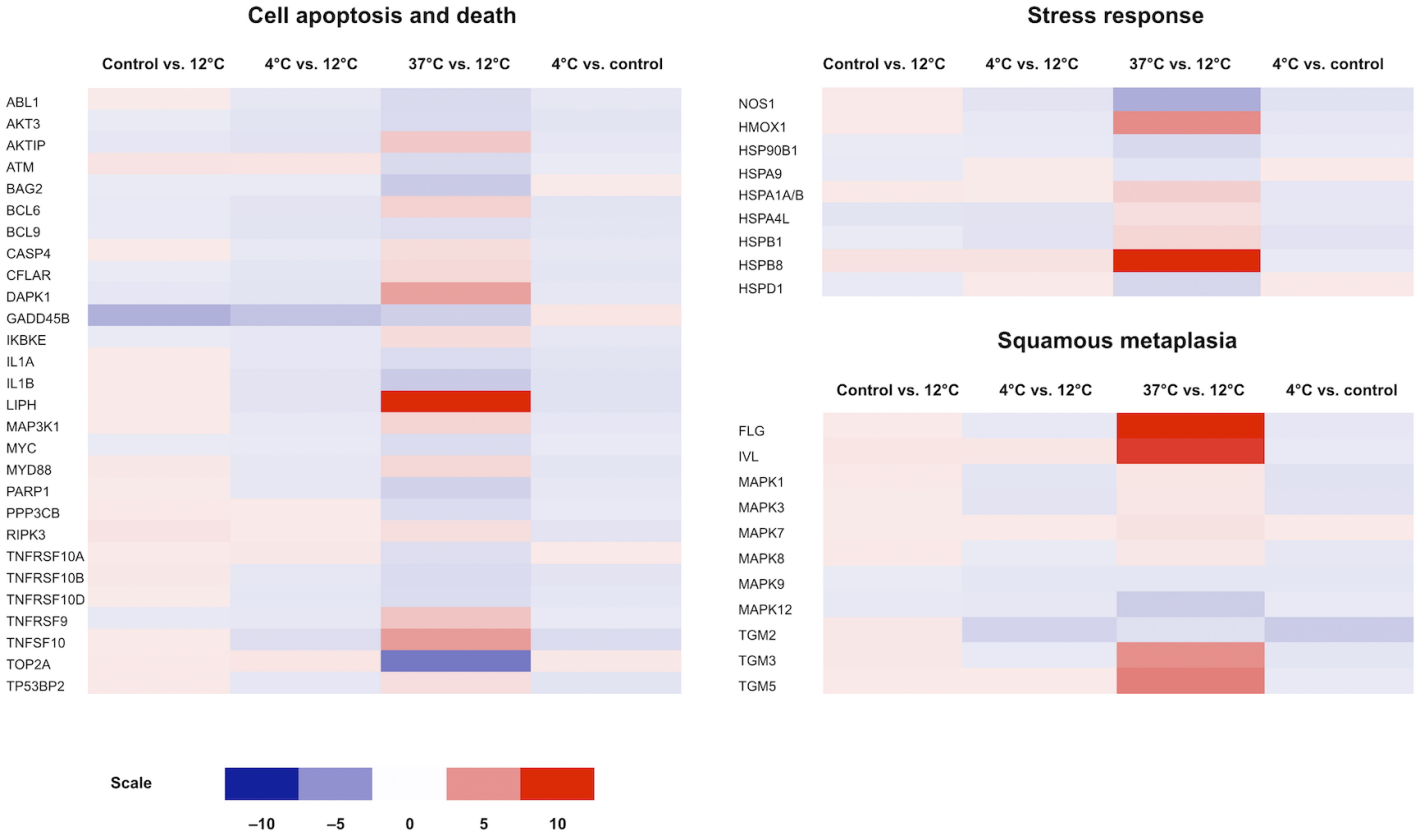
Heat map diagrams of a selection of the most important genes expressed by cultured human oral keratinocytes (HOK) related to cell apoptosis and death, stress response, and squamous metaplasia, respectively. The color scale illustrates the relative expression level of mRNAs: red color represents a high expression level; blue color represents a low expression level.

Very few genetic markers of the oxidative stress response were significantly altered when comparing cells stored at 12°C and 37°C, indicating little difference in oxidative insult between these temperatures (Fig 5). The heat shock protein family members comprise an important cellular defense pathway [37]. The heat shock protein encoding gene HSPB8 was upregulated 27.6-fold at 37°C compared to 12°C, which might indicate cell stress (Table 4). Cornulin, also known as squamous epithelial heat shock protein 53, was upregulated 43.2-fold at 37°C (Table 2). This protein may play a role in the mucosal/epithelial immune response in addition to its role in epidermal differentiation [38]. An additional three heat shock proteins were upregulated between 1.6 and 2.3-fold at 37°C, while two were downregulated 1.8 and 1.9-fold at the same temperature (Table 4).

Various environmental stressors have been shown to induce glucocorticoid production in epidermal keratinocytes [39]. Several genes coding for enzymes, receptors and transport proteins involved in the production of corticosteroids were investigated in the present material. Their expression was either not detected (CYP11B1, CYP11A1, 3βHSD, CYP17, CYP21A2, MC2, StAR) or not significantly altered (NR3C1, encoding the glucocorticoid receptor) when comparing any of the culture groups.

Collectively, these findings suggest that storage at 37°C, compared to 12°C, induces a heat shock response, but does not trigger oxidative stress.

### Regulation of Signaling Pathways

**Fig 6:**
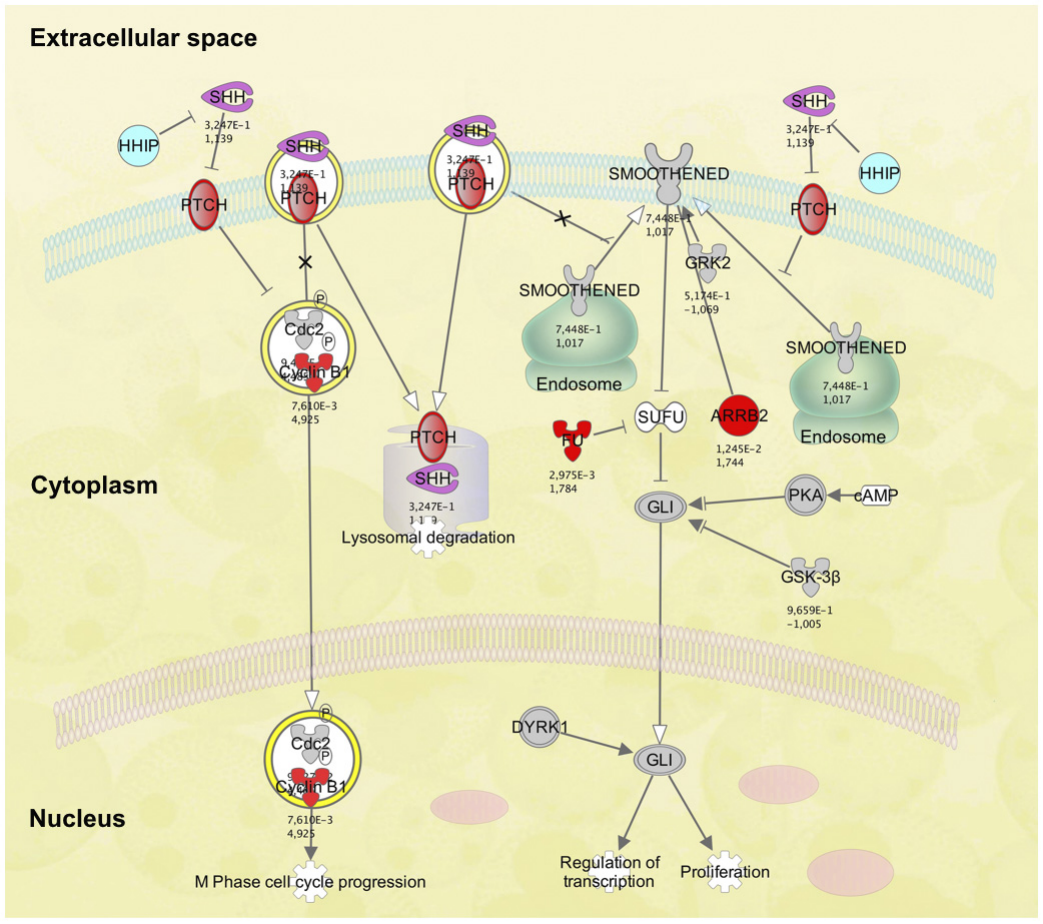
Differential regulation of the Hedgehog signaling pathway at 37°C compared to 12°C. Pathway elements marked in red are significantly downregulated at 37°C. There is no upregulation of pathway elements.

Expression levels of the Wnt, BMP, Hedgehog, JAK/STAT, Notch, and TGF-β signaling pathways were analyzed. Cultures stored at 12°C showed no changes in expression levels of either pathway compared to control cells, and cultures stored at 4°C did not differ from the 12°C cultures. However, cells stored at 37°C expressed slight changes in regulation of various elements of all these pathways compared to the 12°C group. These changes included both up- and downregulation of different elements in all signaling pathways except the Hedgehog pathway. In this pathway, regulation was exclusively negative at 37°C and was comprised of pathway elements cyclin B1 (CCNB1, downregulated 4.9-fold), patched 2 (PTCH2, downregulated 8.6-fold), arrestin, β2 (ARRB2, downregulated 1.7-fold), and serine/threonine kinase 36 (STK36, downregulated 1.8-fold) (Table 4, Figs 4 and 6).

### Regulation of Proliferation, Apoptosis, and Cell Death

The effect of storage temperature on the expression of proliferation and cell death markers was also studied. The proliferation marker Ki-67 [18] was upregulated 7.9-fold at 12°C compared to 37°C cultures, indicative of increased proliferative potential (not shown). Expression of proliferation markers ABCG2 and PCNA was not significantly altered between culture groups (not shown). Slight regulatory changes in the expression of cell death markers were noted between the culture groups, but few genes were markedly changed. Thirteen cell death-related genes were upregulated between 1.6 and 13.7-fold at 37°C compared to 12°C, while 15 genes were downregulated between 1.5 and 6.1-fold (Table 4, Fig 5). Caspase 4 (CASP4) was the only caspase-encoding gene to be differentially expressed, with a 1.7-fold upregulation at 37°C compared to 12°C. Lipase H (LIPH), which is selectively upregulated in lung cancer and associated with increased survival in lung cancer patients [40], was upregulated 13.7-fold at 37°C compared to 12°C.

Of note, of the 114 genes important for cellular function that were significantly regulated at 37°C compared to 12°C (Table 4), only one was differentially regulated when comparing 4°C storage to 12°C storage (Growth arrest and DNA-damage-inducible beta (GADD45B); downregulated 2.81-fold), control cultures to 12°C storage (GADD45B; downregulated 3.6-fold), and 4°C storage to the control (member RAS oncogene family (RAB3B); downregulated 1.53-fold). GADD45 is induced by environmental stress or DNA damage [41], while RAB3B localizes to tight junctions where it has been suggested to contribute to the polarization of epithelia [42, 43]. Hence, 4°C and 12°C storage do not induce notable changes in the regulation of the important genes analyzed herein, and the two storage groups seem to offer equivalent results.

### Quantitative Real-Time PCR Validation of Microarray Data

**Fig 7:**
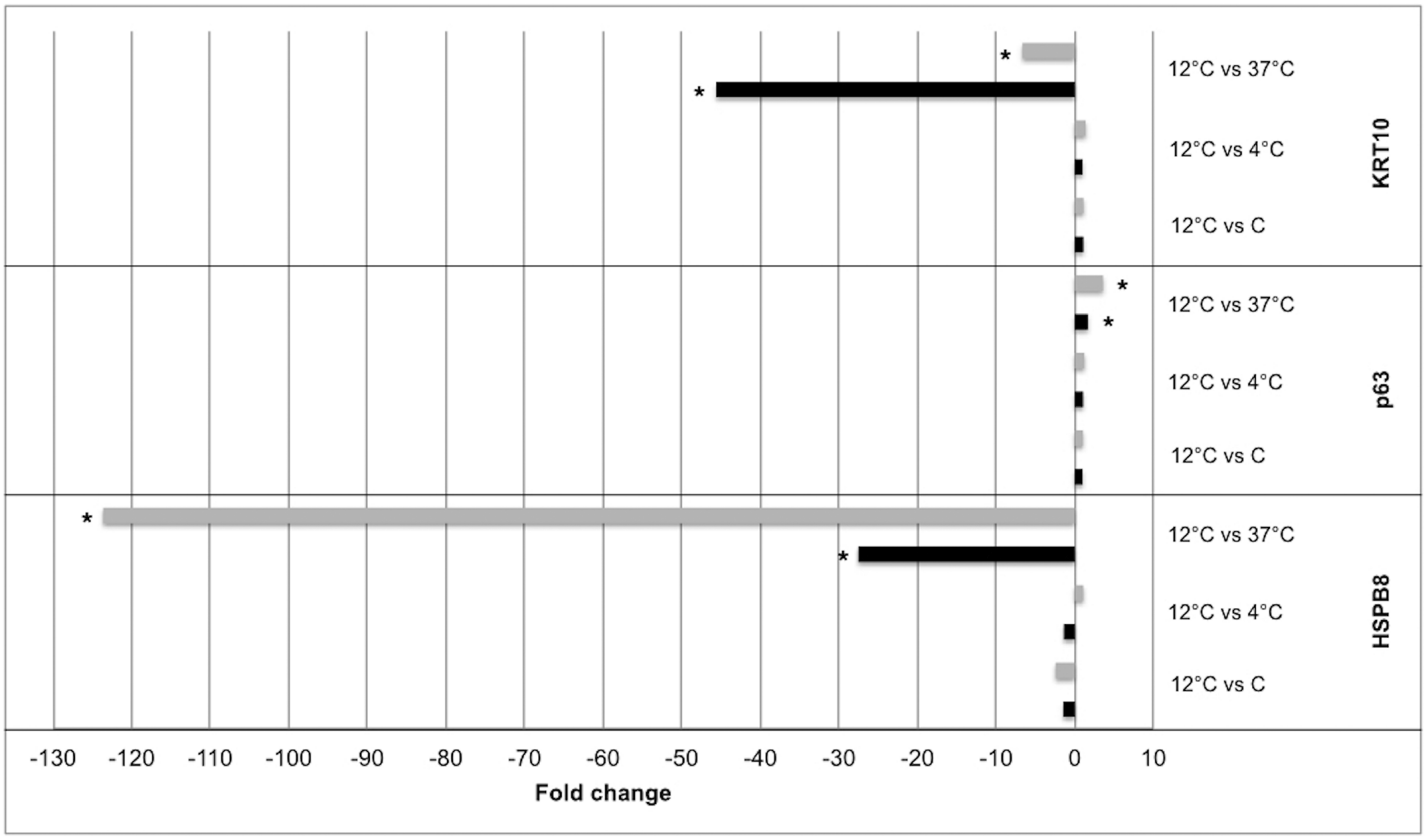
Validation of microarray expression results by qPCR. Selected mRNAs (HSPB8, TP63 and KRT10) were differentially expressed in cultured RPE cells stored at 12°C compared to cultures that were stored at 37°C. Black bars indicate microarray expression values; grey bars represent PCR verification values. *P < 0.05.

HSPB8, TP63 and KRT10 were selected for validation by qPCR (Table 5). The expression of HSPB8 was substantially upregulated at 37°C compared to 12°C; a 123.6-fold upregulation by PCR compared to a 27.6-fold upregulation by microarray. The expression of TP63 was significantly downregulated 3.6-fold at 37°C compared to 12°C, which is in line with the microarray results demonstrating a 1.7-fold downregulation at 37°C. Keratin 10 expression was significantly upregulated 6.7-fold at 37°C compared to 12°C. Upregulation of this gene was higher in the microarray analysis (45.6-fold). Expression levels of HSPB8, TP63, and KRT10 at the remaining temperatures were consistent between qPCR and microarray results, showing no significant differential regulation between temperatures (Table 5 and Fig 7).

**Table 5:** Validation of microarray results by qPCR.

Gene	Affymetrix		PCR	
	Fold Change	P-value	Fold Change	P-value
**HSPB8**				
Control vs 12°C	1.45	0.25	2.45	0.13
4°C vs 12°C	1.38	0.31	-1.09	0.47
37°C vs 12°C	27.55	3.64E-06	123.63	1.00E-03
**TP63**				
Control vs 12°C	1.00	1.00	-1.05	0.30
4°C vs 12°C	-1.07	0.57	-1.22	0.10
37°C vs 12°C	-1.67	1.49E-03	-3.6	5.00E-04
**KRT10**				
Control vs 12°C	-1.09	0.87	-1.12	0.06
4°C vs 12°C	-1.01	0.99	-1.38	0.08
37°C vs 12°C	45.63	9.32E-05	6.67	0.04

## Discussion

The current study investigated the effects of storage temperature on gene expression in cultured HOK using microarray analysis. The temperatures selected included 4°C, the standard temperature of a refrigerator; 12°C, a temperature which previously gave the most optimal results with regard to both morphology and viability of stored HOK; and 37°C, the temperature of a standard cell culture incubator.

Five of the six most differentially regulated proteins at 37°C compared to 12°C are directly associated with epithelial differentiation. An epidermal differentiation profile of these HOK cells is regarded as a disadvantage when used for treating LSCD [9]. The RPTN gene is very active during the final steps of epidermal keratinocyte differentiation, since the repetin protein is associated with the keratin intermediate filaments that are present in mature epidermal cells [14]. Keratinocyte differentiation-associated protein localizes to the stratum corneum of normal skin, but is expressed in suprabasal keratinocytes in psoriatic lesions [17]. Expression of the KDAP gene is markedly upregulated during keratinocyte differentiation *in vitro* [17]. Filaggrin contributes to the hydration and pH homeostasis of the stratum corneum [28], and mutations of the filaggrin gene are associated with ichtyosis vulgaris [44] and eczema [45]. Cornulin is a squamous cell-specific polypeptide [37] which is downregulated in eczema [46] and is a component of the epithelial innate immune response [38]. This protein is a constituent of the heat shock response of esophageal squamous epithelial tissue, where it is known as the squamous epithelial heat shock protein 53 (SEP53) [37]. Cornulin’s expression increases markedly as a consequence of heat shock, which might indicate activation of this cellular defense pathway in cell cultures stored at 37°C compared to those stored at 12°C. However, the activation of cornulin as a component of the differentiation process, and not primarily as a heat shock modulating protein, is an alternative interpretation.

The oral mucosal marker keratin 6b [26] was upregulated 2.5-fold in cultures stored at 37°C compared to 12°C. Its relative downregulation in 12°C cultures might indicate a dedifferentiation of cells at these temperatures. The upregulation of keratin 6b has also been demonstrated in conjunctival epithelium of patients with Sjögren’s syndrome [47]. Keratin 6b is also used as a marker of activated keratinocytes, and its expression can be induced both by proliferative signals and the proinflammatory cytokine TNF-α [48]. TNF was upregulated 1.4-fold at 37°C compared to 12°C (not shown), and several TNF-related proteins are differentially regulated at 37°C (Table 4). Given the stable expression of proliferative markers at 37°C compared to 12°C, it is more likely that the keratin 6b upregulation might be a consequence of inflammatory responses rather than proliferative signals.

CSRNP1 was upregulated 2.4-fold after storage at 12°C compared to control cultures. CSRNP1 has been described as a tumor suppressor gene, its expression level decreased in several types of cancers [49]. Overexpression has been reported to halt cell cycle progression at mitosis [50]. Its function is essential for normal development of the brain [51], and it is a known negative regulator of the Wnt pathway [52].

Both adhesion and tight junction-related genes were significantly upregulated at 37°C compared to 12°C and control cultures. The upregulation of DSC1 in cultures stored at 37°C is in line with our findings in retinal pigment epithelial cells [53]. These findings indicate a functional change towards a more tightly woven, squamous-like epithelium after storage at 37°C.

The expression of stress-related genes was also evaluated. Except for a few heat shock proteins, few genetic markers of the oxidative stress response were significantly altered when comparing cells stored at 12°C and 37°C. Recent studies have described that epidermal keratinocytes subjected to various environmental stressors can respond by upregulating their glucocorticoid production [39, 54]. Expression of genes encoding enzymes, receptors and transport proteins necessary for the production and effect of corticosteroids were either not detected or were unaltered in our material.

Cultures stored at 37°C show both up- and downregulation of several important molecules of the Wnt, BMP, JAK/STAT, Notch, and TGF-β signaling pathways, rendering the effects of these regulational changes inconclusive. However, the Hedgehog pathway was exclusively downregulated in the 37°C cell group compared to the 12°C group. The Hedgehog signaling pathway is instrumental for vertebrate embryogenesis and has been demonstrated to regulate cell fate, proliferation, and survival in multiple cell types, especially those of neuroectodermal origin such as cells of the retina and optic nerve [55]. The pathway also regulates adult stem cells in several self-renewing organs including the subventricular zone of the brain [56, 57]. The deactivating effect of 37°C on this pathway may therefore perturb cellular function. The sonic hedgehog protein, Shh, binds Patched, a transmembrane receptor of the target cell [58]. Patched functions as a tumor suppressor in the hedgehog signaling pathway [58] and mutations of the gene have been detected in basal cell carcinomas and medulloblastomas, among other cancers [55, 59]. The downregulation of Patched in the 37°C culture group compared to 12°C might destabilize cellular quiescence, an undesirable event in cell preservation.

The patched protein interacts with cyclin B1 and participates in determining its cellular localization [60]. Cyclin B1 is a regulatory protein involved in the promotion of mitosis, transitioning the cell from the G2 to M phase. The effect of Patched on Cyclin 1 is inhibition of cellular proliferation [60]. Arrestin β2, which was downregulated at 37°C, is a ubiquitously distributed protein with a critical role in the regulation of several important signaling pathways, including Hedgehog [61]. The serine/threonine kinase 36 (STK36), also downregulated at 37°C, plays a key role in the Hedgehog signaling pathway. Hence, several crucial constituents of the pathway are downregulated at 37°C, contributing to a reduced activity of the pathway as a whole. The finding of perturbed signaling pathway elements of several major pathways in cultures stored at 37°C is in concordance with findings of similar changes in retinal pigment epithelial cells stored at 37°C (unpublished data).

The analysis of cell proliferation and death markers after storage at different temperatures indicates a disturbance of the regulation of several cell death-related genes at 37°C. These changes may be partly responsible for the reduced viability found in cultures stored at this temperature.

Expression of the melatonin receptor MTNR1A was significantly upregulated at 12°C compared to the other groups. Melatonin exerts numerous effects on a multitude of organ systems, including the skin. Its effects are mediated through both receptor dependent and independent mechanisms [62]. Importantly, the skin cannot be regarded a passive target for the effects of melatonin, but a vibrant site of its synthesis and metabolism [63, 64]. There is accumulating evidence that the stringently regulated effects of melatonin in the skin are organized through an interlaced local neuroendocrine system exploiting both auto- and paracrine mechanisms [63, 64]. Melatonin and its metabolites exert broad antioxidant effects, and melatonin is able to activate cytoprotective molecules and enzymes, including glutathione [63, 65, 66]. It may also protect DNA from oxidative damage, thereby providing anti-apoptotic and anti-carcinogenic effects [63, 64, 67]. Specifically, both the initiation and promotion of skin carcinogenesis can be decreased by melatonin [68]. There is also evidence that its oncostatic effects are dependent on the MTNR1A receptor [69–71], and that the tumor suppressive effect of the MTNR1A gene is silenced in oral squamous cell carcinoma [23]. A significant association between MTNR1A polymorphisms and oral carcinogenesis has been demonstrated [22], in which environmental factors (betel quid chewing and cigarette smoking) are required to increase the susceptibility to oral cancer in individuals with MTNR1A gene polymorphisms.

It has also been demonstrated that melatonin and its metabolites protect keratinocytes from UV radiation [64, 72]. Following UVB exposure, MTNR1A expression has been shown to be upregulated in normal neonatal epidermal melanocytes and downregulated in melanoma lines [73]. In the current study, HOK were cultured in the dark except when being handled, but cultures stored at 12°C and 37°C were probably exposed to small amounts of light during heating of the storage containers. This light exposure might have contributed in inducing MTNR1A expression in the 12°C group, but it does not explain why a similar upregulation in the 37°C storage group could not be detected.

While there are clear indications to the upregulation of differentiation-related genes at 37°C compared to 12°C, the gene regulation changes in the 4°C and 12°C groups compared to the control are not as clear. However, the downregulation of some constituents of the epidermal differentiation complex at 4°C might indicate some degree of de-differentiation in these cultures, contrary to the effect of 37°C storage. For cells stored at 12°C, the evidence toward a less differentiated phenotype was not as strong.

In conclusion, HOK cultures stored at 37°C demonstrated considerably larger changes in both the amount of genes affected as well as their differential regulation levels compared to unstored cells than cultured HOK stored at 4°C and 12°C. Temporary storage of cell cultures in sealed containers at 37°C, rather than at 12°C, appears to promote differentiation similarly to conventional cell culture, which employs a humidified incubator at 37°C with CO_2_ supply. Storage at 37°C may reduce the stemness, and thereby the therapeutic potential, of cultured cells intended for the treatment of LSCD [9]. Storage at 12°C also maintains the regulation of genes closer to control levels than storage at 4°C. However, storage at 4°C might steer cells toward a less differentiated phenotype. Nevertheless, we conclude that storage at 4°C and 12°C are more suitable than storage at 37°C for preserving cultured HOK for transplantation. Our findings are in line with a recent study [12], demonstrating superior viability of HOK when stored at 12°C compared to 4°C and 37°C. Thus, collectively, 12°C seems to be the most ideal storage temperature among those investigated.
